# Nutrition literacy and diet quality among adolescents in North Cyprus: a school-based cross-sectional study

**DOI:** 10.1186/s41043-026-01304-y

**Published:** 2026-03-31

**Authors:** Deniz Bolçocuk, Metin Saip Sürücüoğlu

**Affiliations:** Department of Nutrition and Dietetics Faculty of Health Sciences, Cyprus International University, Nicosia, Cyprus

**Keywords:** Adolescence, Nutrition literacy, Diet quality

## Abstract

**Background:**

Nutrition literacy has been increasingly recognized as a fundamental determinant of healthy eating behaviors during adolescence. Adolescence is a critical phase for growth and adoption of long-term healthy habits. However, there are limited evidence on the correlation between nutrition literacy and diet quality in adolescents. This study aims to evaluate the nutrition literacy and diet quality in adolescents and analyze the relationship between these variables.

**Methods:**

This cross-sectional study is comprised of a total of 320 adolescents studying in grade 9–12. Participants were recruited from all state-run secondary schools in the Morphou district using a multi-stage proportional stratified random sampling approach, with proportional allocation across grade and sex strata and random selection within classrooms. The sociodemographic characteristics were collected through a structured survey. The nutrition literacy was evaluated with the Adolescent Nutrition Literacy Scale (ANLS) and diet quality with the Mediterranean Diet Quality Index in Children and Adolescents (KIDMED). Body mass index (BMI) was calculated with anthropometric measurements and BMI percentiles were identified as per age and gender. A correlation analysis was developed to analyze the relationship between the components and nutrition literacy and diet quality scores.

**Results:**

The average age of participants is 15.39 ± 1.10. The average ANLS total score is 69.17 ± 4.75, which indicates a medium-high nutrition literacy. There is no significant different across genders as per their total nutrition literacy scores (*p* = 0.336). Functional nutrition literacy (FNL) has a strong but negative correlation with the interactive nutrition literacy (INL) (*r* = − 0.453; *p* < 0.01). Moreover, FNL reflected weak yet positive correlation with total nutrition literacy (*r* = 0.222; *p* < 0.01) and KIDMED scores (*r* = 0.118; *p* < 0.05).

**Conclusion:**

No significant association was observed between the ANLS total score and diet quality among adolescents. However, a weak but statistically significant association was observed between FNL and diet quality. Strengthening nutrition literacy through age- and gender-sensitive, school-based, and evidence-based interventions may contribute to healthier dietary behaviors and potential long-term improvements in public health outcomes.

**Supplementary Information:**

The online version contains supplementary material available at 10.1186/s41043-026-01304-y.

## Introduction

Adolescence is a critical period where individuals experience accelerated physical, cognitive and social developments that life-long health behaviors are established [1]. The nutritional habits that are acquired in adolescence directly affect the existing health status of individuals as well as any chronic disease risks in later years [2, 3]. However, the higher exposure to media, social environment and peer influence due to modern life may lead the adolescents to consume more unhealthy food and have insufficient nutrition literacy [5].

Nutrition literacy defines the skills to acquire, assess accurate information on food and nutrition, and adopt such in daily life [6–8]. Individuals with high nutrition literacy may have healthy eating decisions while low nutrition literacy would then cause insufficient and unbalanced eating habits [9]. According to previous research, adolescents with low nutrition literacy have much lower diet quality where they are strongly related with unhealthy eating behaviors [10]. Diet quality is another concept that is closely related with nutrition literacy. Diet quality covers the assessment of consumed food in terms of their diversity, balance and nutrients. High diet quality defines sufficient and balanced eating while low diet quality means insufficient or unbalanced diet [11]. Hence, the assessment of nutrition literacy levels and diet quality among high school students is crucial for the improvement of healthy eating habits. The aim of this study is to identify the nutrition literacy levels and diet qualities of high school students and evaluate the correlation across such variables. To our knowledge, this is the first school-based study to assess nutrition literacy and diet quality among adolescents in North Cyprus.

## Methods

This cross-sectional study was conducted with grade 9–12 students aged 14–18 attending three public high schools in Morphou district of Northern Cyprus. All three state-run secondary schools in the district were included in the study, constituting a census of available public schools in the region. The reference population comprised 977 students enrolled in these schools during the academic year 2021–2022. A minimum sample size of 276 students was determined based on a 95% confidence level and 5% margin of error, stratified by grade and sex. To account for potential non-response, a total of 320 students were ultimately recruited. Within each stratum (grade × sex), the number of students to be sampled was determined proportionally to stratum size. Students were then randomly selected within each classroom using simple random sampling. Data collection was carried out between October and December 2022.

Ethical approval for the study was obtained from the Scientific Research and Publication Ethics Committee of Cyprus International University (CIU) (protocol no: EKK21-22/020/004; August 2, 2022). The permission required for the performance of this study was acquired from the Ministry of National Education. The Helsinki Declaration was adhered to during the study, and the parental consent forms were provided to the female and male volunteer adolescents if they fulfilled the study eligibility criteria. Being an adolescent between the age of 14–18, agreeing to participate the study, having Turkish as native language, being healthy are considered as the inclusion criteria while being under the age of 14 and over 18, not agreeing to participate the study and having chronic diseases are recognized as the exclusion criteria.

### Data collection

The written informed consent forms were delivered to the parents via the volunteer students, which the parents signed the next day. The data were collected through face-to-face interview by using questionnaire. The questionnaire has three sections. The English version of the questionnaire is provided as Supplementary File 1. The first section is related with the general information and family details (age, gender, high school, grade, parental education level etc.), eating habits (number of main meals, daily water intake etc.), physical activity level (physical activity, sleep duration and screen time) and anthropometric measurements (weight and height). The second section covers the Adolescent Nutrition Literacy Scale (ANLS) for the analysis of nutrition literacy among adolescents [12], and the final section presents the Mediterranean Diet Quality Index in Children and Adolescents (KIDMED) to evaluate diet quality [13]. The questionnaire was administered in Turkish.

### Adolescent nutrition literacy scale (ANLS)

The nutrition literacy of adolescents was evaluated with the ANLS developed by Bari [4], which was then adopted to Turkish by Türkmen et al. [12]. The scores that can be obtained from the five-point Likert-type scale with twenty-two items range from 22 to 110. The scale has three sub-dimensions as Functional Nutrition Literacy (FNL) (7 items, 7–35 points), Critical Nutrition Literacy (CNL) (9 items, 9–45 points) and Interactive Nutrition Literacy (INL) (6 items, 6–30 points). Higher scale scores shall mean higher levels in nutrition literacy. The Cronbach’s alpha coefficient for the total scale score was 0.80, while the alpha values for the subscales ranged from 0.66 to 0.84. Higher scores obtained from the scale indicate higher levels of nutritional literacy among individuals [12]. In the present study, internal consistency of the ANLS was evaluated using Cronbach’s alpha coefficient.

### Mediterranean diet quality index in children and adolescents (KIDMED)

KIDMED questionnaire, which was developed by Serra Majem et al. [13] to measure the diet adherence among the ages of 2–24, was applied to identify the diet quality in adolescents. The scale has sixteen “Yes” or “No” questions. The index consists of twelve positive and four negative items. A ‘Yes’ response to positive items is scored as + 1 point, whereas a ‘Yes’ response to negative items is scored as − 1 point. A total score of “8 and above (≥ 8 points) is recognized as Optimal (Good) Diet Quality”, “4–7 points - Needs Improvement” (Average), “3 and below (≤ 3 points) – Very Low Diet Quality (Low)”. In the original study, the scale demonstrated acceptable internal consistency, with a Cronbach’s alpha coefficient of 0.69 [13]. Since the KIDMED index consists of dichotomous items, internal consistency in the present study was assessed using the Kuder–Richardson 20 (KR-20) coefficient.

### Anthropometric measurements and evaluation

Researchers took height and weight measurements of students to evaluate Body mass index (BMI) per age and gender. Students took off their excessive clothing and shoes for better data collection during measurements. A digital scale (Tanita BC-730) was used for weight measurement and a non-elastic measuring tape for height. Height measurement was taken in standing upright position on the Frankfort plane, feet close together and head in anatomical position and the percentile values were calculated per age and gender with World Health Organization (WHO) ANDRO PLUS application using BMI percentile curves specific to age and gender. In consideration with BMI percentile classification for ages 5–19, <3rd percentile is classified as risk of underweight, 3rd-15th percentile as underweight, 15th-85th percentile as normal weight, 85th-97th percentile as slightly overweight, and > 97th percentile as obese [14].

### Statistical analysis

Frequency and percentage for qualitative variables; arithmetic mean and standard deviation values for quantitative variables were calculated as descriptive statistics. Normality condition was checked with Kolmogorov-Smirnov test. Non-parametric statistical hypothesis tests were applied due to the violation of parametric assumptions. To compare two independent categories of study variables for scale scores, Mann-Whitney U test was applied. For more than two independent categories, Kruskal—Wallis test was performed. In case of statistical significance, Mann-Whitney U test was applied to explore the pairwise differences. Pearson correlation analysis was performed for understanding associations between quantitative variables. All statistical calculations were carried out with R Software (Ver: 4.5.0 (2025-04-11)) on RStudio environment (Ver:2024.12.1 + 563 for macOS). Significance level was accepted as 0.05. Non-parametric tests were applied for group comparisons due to violation of normality assumptions. For bivariate correlation analyses between continuous variables, Pearson correlation coefficients were computed, which is justified given the large sample size (*n* = 320) in accordance with the Central Limit Theorem.

## Results

The demographic (gender and grade), lifestyle (number of daily main meals and regular exercise) and anthropometric (BMI classification) characteristics of the 320 participants are summarized in Table 1. The study sample consisted of a relatively balanced distribution of males (51.2%, *n* = 164) and females (48.8%, *n* = 156). Participants were distributed across 9th grade (28.7%), 10th grade (26.2%), 11th grade (24.7%), and 12th grade (20.3%). Regarding lifestyle habits, the vast majority of participants (82.8%, *n* = 265) reported consuming three main meals per day. However, regular exercise was uncommon, with an overwhelming majority (96.6%, *n* = 309) stating they do not exercise regularly. Based on age- and sex-specific BMI percentiles, just over half of the participants (53.4%, *n* = 171) were classified as having a healthy weight. A substantial proportion of the sample was classified as either overweight (34.7%, *n* = 111) or having obesity (11.2%, *n* = 36), while a very small percentage (0.6%, *n* = 2) were classified as underweight.

**Table 1:** Demographic, lifestyle, and anthropometric characteristics of the participants

Variables		Total (*n* = 320)	
		n	%
Gender	Male	164	51.2
	Female	156	48.8
Grade	9th	92	28.7
	10th	84	26.2
	11th	79	24.7
	12th	65	20.3
Number of daily main meals	Twice	55	17.2
	Three Times	265	82.8
Regular exercise	Yes	11	3.4
	No	309	96.6
Body mass index classification (Percentile based on age and sex)	Underweight	2	0.6
	Healthy Weight	171	53.4
	Overweight	111	34.7
	Obesity	36	11.2

Descriptive statistics for age, ANLS total and subscale scores, and KIDMED scores are shown in Table 2. The mean age of the participants was 15.39 ± 1.10 years. The mean total ANLS score among the participating adolescents was 69.17 ± 4.75. The mean scores for the subdimensions were 26.58 ± 2.56 for FNL, 17.24 ± 3.21 for INL, and 25.35 ± 2.81 for CNL. The mean KIDMED score of the participants was 4.99 ± 0.63. According to the KIDMED adherence categories, the majority of participants fell into the ‘Needs Improvement’ category (*n* = 310, 96.9%), while 7 participants (2.2%) were classified as having very low diet quality and only 3 participants (0.9%) achieved optimal diet quality.

**Table 2:** Age, Adolescent Nutrition Literacy Scale Scores (Total and Subscales), and KIDMED Scores of the Participants

Variables	Mean (x̄)	SD
Age	15.39	1.10
Adolescent nutrition literacy scale total score - Functional nutrition literacy - Interactive nutrition literacy - Critical nutrition literacy	69.17 26.58 17.24 25.35	4.75 2.56 3.21 2.81
KİDMED Scores	4.99	0.63

The comparison of nutrition literacy and KIDMED scores across different participant characteristics is presented in Table 3. When analyzed by sex, a statistically significant difference was observed only for the FNL score, with females (26.82 ± 2.59) scoring slightly higher than males (26.36 ± 2.52) (*p* = 0.026). No significant differences were found between sexes for INL, CNL, the ANLS Total score, or the KIDMED score (all *p* > 0.05).

Analysis by class grade revealed several significant differences. For FNL, 11th graders (27.04 ± 2.38) scored significantly higher than 10th graders (25.89 ± 2.34) (*p* = 0.019). INL scores were significantly higher for 10th graders (18.10 ± 3.11) compared to 9th graders (16.73 ± 3.19) and 11th graders (16.49 ± 2.99) (*p* = 0.002). For CNL, 11th graders (24.63 ± 2.25) scored significantly lower than 10th (26.01 ± 3.10) and 12th graders (25.86 ± 2.55) (*p* = 0.005). The ANLS Total score was significantly higher for 12th graders (70.55 ± 5.05) compared to both 9th (68.32 ± 4.31) and 11th graders (68.16 ± 4.34) (*p* = 0.004). No significant differences were found among classes for the KIDMED score (*p* = 0.256).

Lifestyle factors, including the number of daily main meals and regular exercise, were not found to be associated with any statistically significant differences in the scores of the ANLS, its sub-dimensions, or the KIDMED score (all *p* > 0.05). The analysis based on BMI classification (percentile-based) yielded significant findings. While FNL and KIDMED scores did not differ significantly across BMI groups (*p* = 0.642 and *p* = 0.600, respectively), other literacy scores showed marked differences. The INL score was significantly lower in the obesity group (15.31 ± 2.81) compared to both the underweight/healthy weight group (17.44 ± 3.30) and the overweight group (17.57 ± 3.00) (*p* < 0.001). Similarly, the CNL score was significantly lower in the obesity group (23.58 ± 2.35) compared to the other two groups (underweight/healthy: 25.84 ± 2.72; overweight: 25.15 ± 2.86) (*p* < 0.001). Consequently, the ANLS Total score was also significantly lower for the obesity group (65.78 ± 3.15) in comparison to both the underweight/healthy weight group (69.91 ± 4.72) and the overweight group (69.13 ± 4.76) (*p* < 0.001).

According to maternal education level, adolescents whose mothers had completed primary school had mean scores of 26.11 ± 2.26 for FNL, 17.34 ± 3.06 for IN, 26.25 ± 2.65 for CNL, a total ANLS score of 69.70 ± 4.58, and a KIDMED score of 4.83 ± 0.64. Among adolescents whose mothers had completed middle or high school, the corresponding mean scores were 26.85 ± 2.58, 17.17 ± 3.49, 25.30 ± 2.79, 69.33 ± 4.88, and 4.99 ± 0.67, respectively. For those whose mothers had a university degree or higher, the mean scores were 26.48 ± 2.63, 17.29 ± 2.96, 25.02 ± 2.84, 68.79 ± 4.67, and 5.06 ± 0.57, respectively. A statistically significant difference was observed in CNL scores across maternal education levels (*p* = 0.038).

According to paternal education level, adolescents whose fathers had completed primary school had a mean FNL score of 26.34 ± 2.36, an INL score of 17.18 ± 2.86, a CNL score of 25.77 ± 2.83, a total ANLS score of 69.30 ± 5.17, and a KIDMED score of 4.86 ± 0.67. Among adolescents whose fathers had completed middle or high school, the corresponding mean scores were 26.84 ± 2.57 for FNL, 16.97 ± 3.32 for INL, 25.48 ± 2.67 for CNL, 69.30 ± 4.31 for the total nutrition literacy score, and 5.01 ± 0.64 for the KIDMED score. For adolescents whose fathers had a university degree or higher, the mean FNL score was 26.37 ± 2.61, the INL score was 17.58 ± 3.19, the CNL score was 25.04 ± 2.96, the total nutrition literacy score was 68.99 ± 5.10, and the KIDMED score was 5.01 ± 0.61. No statistically significant differences were observed among the variables according to paternal education level (*p* > 0.05).

**Table 3:** Comparison of Nutrition Literacy and KIDMED Scores by Participant Characteristics

Variables		Functional nutrition literacy	Interactive nutrition literacy	Critical nutrition literacy	Adolescent nutrition literacy scale	KIDMED score
		x̄±SD	x̄±SD	x̄±SD	x̄±SD	x̄±SD
**Gender**	**Male**	26.36 ± 2.52	17.33 ± 2.90	25.52 ± 3.03	69.21 ± 4.56	5.05 ± 0.62
	**Female**	26.82 ± 2.59	17.15 ± 3.51	25.17 ± 2.56	69.14 ± 4.95	4.92 ± 0.64
***p***		**0.026**	0.407	0.562	0.805	0.131
**Grade**	**9th**	26.60 ± 2.79	16.73 ± 3.19	24.99 ± 2.99	68.32 ± 4.31	5.05 ± 0.69
	**10th**	25.89 ± 2.34	18.10 ± 3.11^**a**^	26.01 ± 3.10	70.00 ± 4.99	4.89 ± 0.47
	**11th**	27.04 ± 2.38^**b**^	16.49 ± 2.99^**b**^	24.63 ± 2.25^**b**^	68.16 ± 4.34	5.00 ± 0.51
	**12th**	26.91 ± 2.57	17.78 ± 3.34	25.86 ± 2.55^**c**^	70.55 ± 5.05^**a**,** c**^	5.02 ± 0.84
***p***		**0.019**	**0.002**	**0.005**	**0.004**	0.256
**Number of daily main meals**	**Twice**	27.18 ± 2.67	16.91 ± 2.89	24.96 ± 2.53	69.05 ± 4.08	5.02 ± 0.45
	**Three times**	26.46 ± 2.52	17.31 ± 3.28	25.43 ± 2.87	69.20 ± 4.88	4.98 ± 0.66
***p***		0.083	0.500	0.224	0.985	0.518
**Regular exercise**	**Yes**	25.55 ± 2.30	18.18 ± 2.32	25.82 ± 3.09	69.55 ± 4.37	4.91 ± 0.30
	**No**	26.62 ± 2.56	17.21 ± 3.24	25.33 ± 2.81	69.16 ± 4.76	4.99 ± 0.64
***p***		0.118	0.252	0.679	0.679	0.579
**Body mass index classification** **(Percentile based** **on age and sex)**	**Underweight or Healthy weight**	26.64 ± 2.44	17.44 ± 3.30	25.84 ± 2.72	69.91 ± 4.72	5.04 ± 0.69
	**Overweight**	26.41 ± 2.63	17.57 ± 3.00	25.15 ± 2.86^**x**^	69.13 ± 4.76	4.93 ± 0.52
	**Obesity**	26.89 ± 2.90	15.31 ± 2.81^**x**,** y**^	23.58 ± 2.35^**x**,** y**^	65.78 ± 3.15^**x**,** y**^	4.94 ± 0.63
***p***		0.642	**< 0.001**	**< 0.001**	**< 0.001**	0.600
**Education of mother**	**Primary School**	26.11 ± 2.26	17.34 ± 3.06	26.25 ± 2.65	69.70 ± 4.58	4.83 ± 0.64
	**Secondary & high school**	26.85 ± 2.58	17.17 ± 3.49	25.30 ± 2.79	69.33 ± 4.88	4.99 ± 0.67
	**University degree or above**	26.48 ± 2.63	17.29 ± 2.96	25.02 ± 2.84^**k**^	68.79 ± 4.67	5.06 ± 0.57
***p***		0.178	0.831	**0.038**	0.442	0.161
**Education of father**	**Primary school**	26.34 ± 2.36	17.18 ± 2.86	25.77 ± 2.83	69.30 ± 5.17	4.86 ± 0.67
	**Secondary & high school**	26.84 ± 2.57	16.97 ± 3.32	25.48 ± 2.67	69.30 ± 4.31	5.01 ± 0.64
	**University degree or above**	26.37 ± 2.61	17.58 ± 3.19	25.04 ± 2.96	68.99 ± 5.10	5.01 ± 0.61
***p***		0.357	0.228	0.213	0.743	0.622

A: Statistically significant difference from 9th graders

b: Statistically significant difference from 10th graders

c: Statistically significant difference from 11th graders

x: Statistically significant difference from underweight and healthy weight group

y: Statistically significant difference from overweight group

k: Statistically significant difference from primary school group

*p* < 0.05 statistically significant

KIDMED, Mediterranean Diet Quality Index in Children and Adolescents

The bivariate correlations between nutrition literacy scores, KIDMED score, and key lifestyle and anthropometric variables are presented in Table 4. FNL demonstrated a strong, negative correlation with INL (*r* = -0.453, *p* < 0.01), indicating that as functional literacy scores increased, interactive literacy scores tended to decrease. Conversely, it showed a weak, positive correlation with the ANLS Total score (*r* = 0.222, *p* < 0.01) and the KIDMED Score (*r* = 0.118, *p* < 0.05). Functional literacy was also weakly and negatively correlated with daily sleeping hours (*r* = -0.140, *p* < 0.05), monitor hours on weekdays (*r* = -0.135, *p* < 0.05), and monitor hours on weekends (*r* = -0.139, *p* < 0.05).

INL was strongly and positively correlated with CNL (*r* = 0.301, *p* < 0.01) and the ANLS Total score (*r* = 0.611, *p* < 0.01). It also had weak, positive correlations with daily sleeping hours (*r* = 0.135, *p* < 0.05), weekday monitor hours (*r* = 0.274, *p* < 0.01), and weekend monitor hours (*r* = 0.233, *p* < 0.01). CNL showed a very strong, positive correlation with the ANLS Total score (*r* = 0.787, *p* < 0.01). A significant, weak negative correlation was found between CNL and BMI Percentile (*r* = -0.189, *p* < 0.01), suggesting that higher critical literacy is associated with a lower BMI percentile.

The ANLS Total score was positively but weakly correlated with weekend monitor hours (*r* = 0.162, *p* < 0.01) and daily sleeping hours (*r* = 0.128, *p* < 0.05). Importantly, it was also negatively correlated with BMI Percentile (*r* = -0.174, *p* < 0.01), indicating that higher overall nutrition literacy was associated with a lower BMI percentile. Among the other variables, age was positively and moderately correlated with weekend monitor hours (*r* = 0.299, *p* < 0.01). Daily sleeping hours showed a weak, positive correlation with weekend monitor hours (*r* = 0.143, *p* < 0.05). As expected, weekday and weekend monitor hours were moderately and positively correlated with each other (*r* = 0.419, *p* < 0.01). No other statistically significant correlations were observed for the KIDMED Score or age with the other variables. In the correlation analysis, low-level positive associations were observed between daily water intake and FNL (*r* = 0.101), INL (*r* = 0.062), CNL (*r* = 0.012), the total ANLS score (*r* = 0.092), and the KIDMED score (*r* = 0.032). Daily water intake also showed weak associations with age (*r* = − 0.018), daily sleeping hours (*r* = 0.081), weekday monitor hours (*r* = 0.064), weekend monitor hours (*r* = 0.162), and BMI percentile (*r* = 0.002). Among these variables, only the association between daily water intake and weekend monitor hours was statistically significant (*p* < 0.01). Internal consistency for ANLS was assessed using Cronbach’s alpha and was found to be α = 0.75. Cronbach’s alpha values for the subscales were 0.39 for FNL (7 items), 0.42 for INL (6 items), and 0.52 for CNL (9 items). For KIDMED, internal consistency was assessed using the KR-20 coefficient due to its dichotomous items and was found to be 0.64 (16 items).

**Table 4:** Relationships between nutrition literacy, KIDMED score, and lifestyle/anthropometric variables

Variables	FNL	INL	CNL	ANLS	KIDMED Score	Age	Daily Sleeping hours	Monitor Hours - Weekdays	Monitor Hours - Weekends	BKI Percentile	Dailly Water Consumption (glass)
**FNL**	1.000	−0.453 **	−0.018	0.222 **	0.118 *	0.089	−0.140 *	−0.135 *	−0.139 *	−0.049	0.101
**INL**		1.000	0.301 **	0.611 **	−0.041	0.041	0.135 *	0.274 **	0.233 **	−0.052	0.062
**CNL**			1.000	0.787 **	−0.037	0.023	0.034	0.083	0.076	−0.189 **	0.012
**ANLS**				1.000	0.014	0.089	0.036	0.162 **	0.128 *	−0.174 **	0.092
**KIDMED Score**					1.000	0.032	−0.001	−0.081	−0.093	−0.080	0.032
**Age**						1.000	−0.064	−0.073	0.299 **	−0.069	-0.018
**Daily sleeping hours**							1.000	0.075	0.143 *	0.002	0.081
**Monitor Hours - Weekdays**								1.000	0.419 **	−0.007	0.064
**Monitor Hours - Weekends**									1.000	−0.039	0.162 **
**BKI Percentile**										1.000	0.002
**Dailly Water Consumption (glass)**											1.000

*: *p* < 0.05; **: *p* < 0.01

ANLS, Adolescent Nutrition Literacy Scale; BMI; Body Mass İndex; CNL, Critical Nutrition Literacy; FNL, Functional Nutrition Literacy; INL, Interactive Nutrition Literacy; KIDMED, Mediterranean Diet Quality Index in Children and Adolescents

## Discussion

This study investigated nutrition literacy and diet quality among adolescents and examined the associations between these variables. The findings demonstrated that nutrition literacy levels were relatively high, whereas adherence to the Mediterranean diet was moderate. A weak but statistically significant positive association was observed between functional nutrition literacy and diet quality. The weak association observed between functional nutrition literacy and diet quality may indicate that nutrition knowledge alone may not always translate into healthier dietary behaviors among adolescents. Additionally, total nutrition literacy and certain subdimensions were weakly and negatively associated with BMI percentile. Overall, the observed relationships were weak in magnitude, indicating limited strength of associations between nutrition literacy, diet quality, and anthropometric indicators.

The WHO defines adolescence as a critical phase in life, which covers the transitional period ranging the ages of 10–19 between childhood and adulthood. This phase is one of the most important phases in one’s life development as a determinant in the foundation of future health. Adolescents experience much accelerated physical, cognitive and psychosocial development where they may establish basic behavioral patterns like nutrition and physical activity that may affect the health of their own and surroundings [15].

The improvement of eating habits and diet quality in adolescence introduce long-term effect on the health, chronic disease risk and overall quality of life in adult life. Poor diet quality and inadequate nutrient intake in adolescents are a global public health problem, which may cause an early introduction of micronutrient deficiencies, excess weight and obesity prevalence and cardiometabolic risk factors [5]. Hence, nutrition literacy is a fundamental indicator in the adoption of health eating habits among adolescents. Insufficient nutrition literacy may cause difficulties in meeting the increasing eating needs and lower the diet quality. The increasing number of studies in literature emphasizes the strong correlation between the nutrition literacy and diet quality in adolescents. This study covers a total of 320 students (164 male, 156 female) from grades 9, 10, 11 and 12. The average ages of participants are consistent with the similar studies [16–18], which promotes the comparability of study findings with the adolescent population.

The total nutrition literacy is calculated as 69.17 ± 4.75, which is in parallel with the outputs of many similar studies [19–23]. On the other hand, there also various studies with different scores [24, 25].

In consideration with the sub-dimensions, the functional, interactive and CNL scores are similar with the other studies [19, 22, 23], which indicates the accurate evaluation of multi-dimensional nature of nutrition literacy in adolescents.

According to the analysis based on the gender variable, there is no significant different between overall nutrition literacy and sub-dimension scores, which is compatible with the study results by Koca et al. [26] and other similar studies respectively [27, 28]. On the other hand, there are some studies that report differences between genders particularly on the interactive and CNL [21, 29]. Such contradicting results may indicate that the impact of gender on nutrition literacy is susceptible to the contextual factors.

The findings on the correlation between nutrition literacy and BMI are inconsistent. While some studies reflect the variance of CNL by BMI groups [26], other studies did not conclude any significant relation between overall score and sub-dimensions and BMI [18, 22, 28, 29]. However, pursuant to our study, total, interactive and CNL levels are significantly different by BMI classification without any relation between INL and BMI. This may reflect that the nutrition literacy particularly the critical and practical dimensions may be closely related with body weight.

From the perspective of our study, the nutrition literacy scores by grades are significantly different in all sub-dimensions. Another study noted significant difference only in functional and interactive literacy among the scale sub-dimensions [30] whereas a different study indicated a significant difference in CNL [28], which may be related with the difference in sample, education programs or age distribution. Since there is a limited number of studies regarding the change in nutrition literacy as per age groups and grades, further studies should be conducted accordingly.

In consideration with the relation between the education levels of parents and nutrition literacy, there is no significant relation with parents’ education level and total nutrition literacy. This output is consistent with some studies [27]; however, there are also some studies suggesting the influence by the education level of father [26], which indicates that the family eating environment is not only shaped with the education level, but also together with cultural and behavioral elements.

There are various studies concluding that nutrition literacy scores of individuals with high water intake is high [20]. However, this study did not detect any significant relation between water intake and nutrition literacy, which may be due to the evaluation of water intake via self-reporting and variance in individual lifestyle habits.

In terms of nutrition literacy and KIDMED scores, the relevant scores are low in the sample without any significant relation with the total nutrition literacy. On the other hand, there are also some studies reflecting positive relation between literacy level and KIDMED score [16, 21, 26, 31, 32], which may be due to the differences in eating habits, socioeconomic status, area of residency or school types among students.

Pursuant to our study, 17.2% of participants consume two main meals while 82.8% have three main meals in a day, which is consistent with literature where it is concluded that adolescents mainly consume two or three daily main meals [33, 34]. Moreover, some studies reported that the level of individuals that consume two or less meals are higher and fewer people have four or more main meals daily [35].

It is noteworthy for our study that the rate of adolescents engaging is regular physical activity is very low, which is contradicting with some studies noting high physical activity levels [36]. The absence of significant relation between physical activity and nutrition literacy is consistent with the literature [26], which may mean that physical activity may be much closely related with environmental facilities, individual motivation and habits rather than knowledge.

In the present study, the internal consistency of the ANLS total score was acceptable (α = 0.75), although slightly lower than the original value (α = 0.80). However, subscale reliabilities (0.39–0.52) were lower than previously reported (0.66–0.84) [12]. The KIDMED index showed moderate internal consistency (KR-20 = 0.64), comparable but slightly lower than the original value (α = 0.69) [13]. These differences may reflect variations in sample characteristics and sociocultural context. It should be noted that reliability coefficients may vary across different populations and cultural contexts even when the same measurement instrument is used.

A key strength of this study is that it represents the first population-level evidence on nutrition literacy and diet quality among adolescents in North Cyprus. On the other hand, the main limitations of this study are the mean of data collection as self-reporting and being limited with specific timeframe. Future studies with much comprehensive sample size and multi-centered nature are recommended for much elaborated assessment between the nutrition literacy and eating habits and anthropometric indicators.

## Conclusion and recommendations

In general, the nutrition literacy levels are high but the adherence to the Mediterranean diet is medium level indicating a gap between the compatibility of knowledge and behavior. No significant association was observed between the ANLS total score and diet quality among adolescents; however, a weak but statistically significant association was observed between FNL and diet quality. This outcome underlines that the nutrition literacy and healthy eating habits in adolescents are not always in parallel, and some interventions to promote changes in behaviors may be required respectively. The study results also address the need for gender- sensitive and practical training programs to improve the nutrition literacy in adolescents. Moreover, the dietitians and professionals from the field must have an active role in the planning and execution of school-based nutrition trainings to ensure the scientific foundations and sustainability in such trainings. Additionally, horizontal and experimental studies are also required to reflect the efficiency of such interventions. However, the weak correlations observed in this study should not be over-interpreted as evidence of a strong relationship.

## Limitations

This study bears some limitations where the cross-sectional design does not allow to analyze the time dimension in the relations between nutrition literacy and diet quality and limit the causal inferences. Since this study covers all of three public schools in Morphou, which limits the generalization of results with the adolescents in different districts. The eating habits, details on family and physical activity levels are all evaluated through self-reported data where the availability of physical activity is enquired from yes/no type questions. On the other hand, the researcher directly has taken the anthropometric measurements, which is one of the strengths of this study.

A limitation of this study relates to the internal consistency of the measurement scales. While the ANLS total score demonstrated acceptable reliability, subscale reliabilities were lower than those reported in the original study [12]. Similarly, the internal consistency of the KIDMED index was moderate and slightly lower than previously reported [13].

## Supplementary Information

Below is the link to the electronic supplementary material.

Supplementary Material 1

## Data Availability

All data generated or analyzed within the scope of this study are covered in the published article. Additional data may be obtained from the corresponding author upon reasonable request.

## Abbreviations

ANLS: Adolescent Nutrition Literacy Scale
BMI: Body Mass Index
CIU: Cyprus International University
CNL: Critical Nutrition Literacy
FNL: Functional Nutrition Literacy
INL: Interactive Nutrition Literacy
KIDMED: Mediterranean Diet Quality Index in Children and Adolescents
KR-20: Kuder–Richardson 20 (KR-20)
WHO: World Health Organization
