# Supplementary material for: Nutrition literacy and diet quality among adolescents in North Cyprus: a school-based cross-sectional study
Source: J Health Popul Nutr. 2026 Mar 31;45:146. doi: 10.1186/s41043-026-01304-y (PMC13244904; doi:10.1186/s41043-026-01304-y)
Supplement: Supplementary file 1 — Supplementary Material 1 [file 41043_2026_1304_MOESM1_ESM.docx]

# Supplementary File 1

# Author-developed questionnaire sections (Sections 1)

# This file includes the English version of the questionnaire items developed by the authors for the present study. Previously published scales (ANLS and KIDMED) are not included.

# Nutrition Literacy and Diet Quality Among Adolescents in North Cyprus

**School-Based Cross-Sectional Study Questionnaire**

## Section 1.

## Sociodemographic Characteristics

- Gender: ☐ Female ☐ Male
- Grade: ☐ 9th ☐ 10th ☐ 11th ☐ 12th
- Age: ______

## Anthropometric Measurements

- Body Weight (kg): ______
- Height (cm): ______
- Body Mass Index (BMI): ______

## Lifestyle Characteristics

- Number of Daily Main Meals: ☐ Twice ☐ Three times
- Regular Physical Activity: ☐ Yes ☐ No
- Daily Sleeping Duration: ☐ <6 ☐ 6–7 ☐ 8–9 ☐ ≥10 hours
- Screen Time (Weekdays): ______ hours/day
- Screen Time (Weekends): ______ hours/day
- Daily Water Consumption: ______ glasses/day

## Parental Education Level

- Mother’s Education: ☐ Primary ☐ Secondary & High ☐ University+
- Father’s Education: ☐ Primary ☐ Secondary & High ☐ University+

## Section 2. Adolescent Nutrition Literacy Scale (ANLS)

## Section 3. KIDMED Index
